# On the ecogeomorphological feedbacks that control tidal channel network evolution in a sandy mangrove setting

**DOI:** 10.1098/rspa.2015.0115

**Published:** 2015-08-08

**Authors:** B. van Maanen, G. Coco, K. R. Bryan

**Affiliations:** 1 Faculty of Engineering and the Environment, University of Southampton, University Road, Southampton SO17 1BJ, UK; 2 School of Environment, The University of Auckland, Private Bag 92019, Auckland, New Zealand; 3 Faculty of Science and Engineering, University of Waikato, Private Bag 3105, Hamilton, New Zealand

**Keywords:** tidal channel networks, mangroves, biophysical interactions, numerical modelling

## Abstract

An ecomorphodynamic model was developed to study how *Avicennia marina* mangroves influence channel network evolution in sandy tidal embayments. The model accounts for the effects of mangrove trees on tidal flow patterns and sediment dynamics. Mangrove growth is in turn controlled by hydrodynamic conditions. The presence of mangroves was found to enhance the initiation and branching of tidal channels, partly because the extra flow resistance in mangrove forests favours flow concentration, and thus sediment erosion in between vegetated areas. The enhanced branching of channels is also the result of a vegetation-induced increase in erosion threshold. On the other hand, this reduction in bed erodibility, together with the soil expansion driven by organic matter production, reduces the landward expansion of channels. The ongoing accretion in mangrove forests ultimately drives a reduction in tidal prism and an overall retreat of the channel network. During sea-level rise, mangroves can potentially enhance the ability of the soil surface to maintain an elevation within the upper portion of the intertidal zone, while hindering both the branching and headward erosion of the landward expanding channels. The modelling results presented here indicate the critical control exerted by ecogeomorphological interactions in driving landscape evolution.

## 1. Introduction

Mangroves are highly productive ecosystems that cover the intertidal area of many tropical and subtropical coastlines [1,2]. They have become adapted to a particular environment where only a few other species can compete. In fact, mangroves are the only woody plants occupying the margin between land and sea in low latitudes [3]. Over recent decades, these ecosystems have been highlighted as areas of global importance as mangrove forests provide critical ecosystem services such as carbon sequestration [4], create habitats for a variety of organisms [5] and protect against coastal hazards [6]. Mangrove trees can grow on a variety of substrates, including mud, sand and carbonate sediments [7] and they usually occur in sheltered environments, such as estuaries and embayments. Although sheltered from intense wave action, these environments are dynamic and undergo morphological change as a result of physical feedbacks that involve tidal currents and associated sediment transport [8], mediated by biological agents. The evolution of these tidal systems becomes even more complex, in fact, when mangroves are present as aquatic vegetation has proved to be capable of modifying its physical environment [9]. Although there is no doubt about the ecological and economic value of mangrove ecosystems and the question of how such a highly coupled physical–biological system evolves is of great scientific interest, relatively little research has focused on the interlinked processes that shape the evolution of these ecosystems and the morphological setting that hosts them.

**Figure 1.**
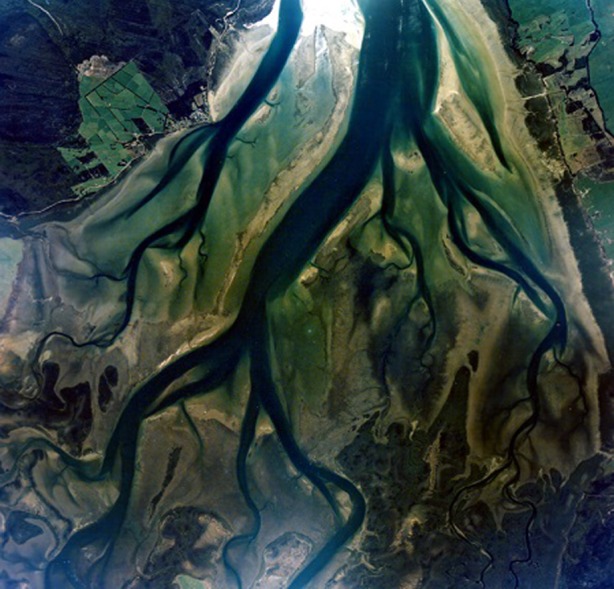
Tidal channel network and mangroves in the Rangaunu Harbour, North Island, New Zealand. Photo courtesy of the Department of Conservation, NZ.

Mangrove forests in estuaries and embayments are often dissected by tidal channels, giving rise to intriguing morphological patterns (figure 1). The channel forming processes resemble those of salt marsh channels; channels typically form because flowing water concentrates within small-scale topographic depressions, leading to an increase in flow velocity and the erosion and deepening of the initial depression [10]. This drives a further concentration of the flow so that a positive feedback mechanism between erosion and channel formation is created [8,11,12]. In addition to this flow-sediment-topography feedback, mangroves are expected to interact with these physical processes and to change the dynamics related to the formation as well as the subsequent evolution of entire tidal channel networks. On the other hand, mangroves are dependent on physical processes as their habitat is restricted to areas with specific salinity and inundation regimes, and hence inherently linked to bed elevation [13]. A two-way biophysical coupling thus exists and it is this type of mutual influence of biotic and abiotic components that can potentially govern large-scale, long-term landscape evolution [9,14,15].

A number of studies have already examined the one-way coupling in which mangrove trees affect hydrodynamic and sediment transport processes. This has been done through both field and laboratory experiments, as well as modelling. Numerical modelling studies of hydrodynamic processes have shown that mangrove trees have a significant impact on the flow structure in mangrove creeks by enhancing the tidal asymmetry [16,17]; this effect was attributed to the extra flow resistance in the mangrove forest (see also [18]). A two-dimensional depth-integrated model that includes both the effects of the drag force and the blockage induced by mangrove trees was developed by Wu *et al.* [19]. They performed modelling exercises with idealized cases as well as a real estuary and found that tidal velocities were significantly reduced in the forested area while an increase in velocities occurred in the main channel. More recently, Horstman *et al.* [20,21] used extensive field measurements and state-of-the-art modelling techniques to study tidal-scale flow routing and associated sedimentation patterns in a mangrove site in Southern Thailand. They concluded that the tidal exchange was dominated by creek flow, although the contribution by sheet flow through the mangrove vegetation increased for the highest tides. During these higher tides, the sheltered interior of the forest also served as an effective sediment sink. This capability of mangroves to trap sediment was also highlighted by Furukawa & Wolanski [22], Furukawa *et al.* [23] and van Santen *et al.* [24]. Related to this enhanced sediment trapping are the field observations described by Stokes *et al.* [25], who showed that the bed elevation decreased by 9–38 mm yr^−1^ in areas where mangroves had been removed, while the surface generally accreted where the mangroves were still intact, emphasizing again the influence of mangroves on sediment dynamics and the importance of better understanding these environments also because of ongoing climatic changes and increasing anthropogenic activities at the shoreline.

The studies described above and other similar types of studies have provided valuable insight into the way mangroves can interact with physical processes. However, the question of how such biophysical interactions affect the morphological evolution of tidal landscapes over decades or even longer time scales has so far been largely unexplored. This includes the influence of mangroves on the formation and evolution of tidal channel networks. These networks control to a large extent the fluxes of water, sediments and nutrients [26,27], and their characteristics determine the efficiency of the network to drain and feed the tidal landscape [28]. As such, tidal networks are critical to the overall functioning of the system. For salt marsh systems, some spatially explicit morphodynamic models incorporating vegetation effects have already been developed [29–32], showing that the evolution of salt marshes cannot be fully understood without considering biophysical interactions.

Here, we present a numerical model which accounts for the ecogeomorphological feedbacks arising from the presence of mangroves, which we have developed to study the role of mangroves in the large-scale and long-term morphological evolution of sandy tidal embayments. The focus is on channel network-forming processes. Such a model requires the incorporation of an ecosystem module which describes mangrove growth as a function of the physical environment and vegetation dynamics. Mangrove species vary somewhat in their trunk and root characteristics, and in this study we consider *Avicennia marina*, a species which occurs in both hemispheres and with a range that extends into cooler warm-temperate climates [33]. The effects of mangroves on physical processes that have been included in the model are: (i) increasing flow resistance; (ii) increasing resistance of sediment to erosion by tidal flow; (iii) increasing resistance of sediment to slope-driven sediment transport; and (iv) soil expansion driven by the production of organic matter. The latter process has received particular interest with respect to the ability of mangrove forests to adjust to sea-level rise [34]. In this context, we performed additional simulations to explore how the channel network might evolve under a rising sea level in both the presence and absence of mangrove vegetation.

## 2. Methodology

We used a numerical model which is capable of simulating the long-term morphological evolution of tidal embayments as a result of the interactions between hydrodynamics, sediment transport and the evolving morphology. This morphodynamic model was coupled to a mangrove-population model so that the interactions between mangroves and physical processes could be explored. The development of the morphodynamic model was originally described by van Maanen *et al*. [35]. It is capable of simulating realistic tidal basin morphologies and the model has been previously used to study the effects of tidal range and initial bathymetry on channel network formation [36] and to simulate the response of tidal embayments to sea-level rise [37]. As such, it provides a good opportunity to extend this model to explore the influence of biophysical feedbacks. As the model has been previously discussed in detail, we will only give a brief description here (see §2a). After that, we explain in detail how we treated the colonization by mangroves, and the growth and mortality of the trees as this is a key element of our modelling approach (see §2b). The effects of mangroves on hydrodynamics and sediment dynamics will be described as well (see §2c). Accounting for biophysical interactions within morphodynamic models is not straightforward as it is not always clear how to represent all processes in a quantitative way [9]. Overall, the model developed here falls in the category of ‘exploratory’ models [38] which are critical to improve our understanding but whose quantitative predictive skills are likely to need future refinements. The parametrizations that we adopted were developed to capture the main dynamics of mangrove environments and are not meant to result in quantitatively accurate predictions. In §2d,e, we describe the model set-up and the technique used here to extract the channel networks from the simulated morphologies.

### (a) Morphodynamic model

The morphodynamic model applies the Estuary and Lake Computer Model (ELCOM; [39]) to obtain information regarding the tidal currents and water depths throughout a tidal cycle. ELCOM is a three-dimensional hydrodynamic model based on the unsteady Reynolds-averaged Navier–Stokes equations. The equations are solved on a rectangular grid. Influences of Coriolis force, density differences, wind and waves are neglected.

The depth-averaged flow fields, which are computed every hydrodynamic time-step, are used to obtain instantaneous sediment transport rates which are calculated according to the sediment transport formula developed by Engelund & Hansen [40]. Slope-driven sediment transport is incorporated by following the approach of Kirwan & Murray [31]. Gradients in sediment transport rate yield bed-level changes due to conservation of sediment mass. To facilitate the execution of long-term simulations, various methods have been developed to increase the rate of morphological change [41]. For the morphodynamic model, we applied here, bed-level changes are first integrated over one tidal cycle and then multiplied by a morphodynamic time-step before feeding back into the hydrodynamic model as an updated bathymetry. This morphodynamic time-step (i.e. a specific number of tidal cycles) is optimized every iteration by increasing it until bed-level changes at any single grid cell within the domain exceed 10% of the local water depth at high tide. Subsequently, the morphodynamic time-step is applied to all grid cells to compute overall morphological change.

### (b) Mangrove colonization, growth and mortality

The upper and lower limits of the distribution of mangroves are governed by inundation patterns, and thus closely related to bed elevation [13]. Clarke & Myerscough [42] found the distribution of *A. marina* to lie in between mean sea level and mean high water. This is simulated by allowing mangroves to establish themselves only in grid cells which are inundated less than half of the time. Mangroves can start growing every year and the probability of initial establishment in a bare grid cell is set to 5%, which is low as the dispersal distance of propagules (germinated seedlings used for reproduction) is generally limited (although the dispersal of propagules further than 10 km has also been observed [43]). Furthermore, mangroves are allowed to expand laterally to the four neighbouring grid cells. The initial mangrove density is set to 3000 individuals per ha (which corresponds to 3000 individuals per grid cell) and the mangroves are given an initial stem diameter of 1.37 cm. These parameter values are taken from Berger & Hildenbrandt [44] who used these settings in their mangrove-population model.

Growth of the mangrove trees is described by following Berger & Hildenbrandt [44], Shugart [45] and Chen & Twilley [46]:

2.1 $$\frac{dD}{dt}=\frac{GD(1-(DH)/(D_{\max}H_{\max}))}{\left(274+3b_{2}D-4b_{3}D^{2}\right)},$$

where *D* is the stem diameter (cm), *H* is the tree height (cm), and *t* is time (years). *D*_max_ and *H*_max_ are the maximum stem diameter and tree height and are set to 40 and 1000 cm, respectively. These mangrove dimensions are based on observations of *A. marina* trees in northern New Zealand [47]. *G*, *b*_2_ and *b*_3_ are species-specific growth parameters which are set here to, respectively, 152.17 cm yr^−1^, 43 and 0.536 cm^−1^ such that the maximum increase in stem diameter is 1 cm yr^−1^. Tree height and stem diameter are related according to the following formulation which assumes that a tree with a diameter of 0 cm is 137 cm in height [44,45]. This is because the stem diameter is usually defined at breast height, which is formalized as a distance of 137 cm (=4.5 feet) above ground level:

2.2 $$H=137+b_{2}D-b_{3}D^{2}.$$

Equation (2.1) defines the increase in stem diameter over time under optimal growth conditions. In reality, however, the growth of mangroves is limited by stresses. We here consider inundation (*I*) and competition (*C*) stress as the main factors controlling mangrove growth and the effects of these stresses are, similar to Chen & Twilley [46] and Berger & Hildenbrandt [44], incorporated by adding correction factors to equation (2.1):

2.3 $$\frac{dD}{dt}=\frac{GD(1-(DH)/(D_{\max}H_{\max}))}{\left(274+3b_{2}D-4b_{3}D^{2}\right)}\cdot I\cdot C.$$

These stress multipliers *I* and *C* range between 0 (no growth) and 1 (unlimited growth). *I* is dependent on the hydroperiod and it is assumed that there is an optimal inundation regime for which the growth rate is at its maximum (*I*=1), with reduced growth rates (*I*<1) when the mangroves are inundated for either longer or shorter. The growth and size of the mangrove trees is thus directly dependent on inundation regime and thus also linked to bed elevation. This type of response of the growth rate of mangroves to flooding has been previously described by, for example, Krauss *et al.* [13]. Competition among mangroves affects growth when neighbouring trees have to share the available resources. The correction factor for competition *C* is thus dependent on the total biomass of the mangrove trees and *C* decreases (reducing mangrove growth) with increasing biomass. Details concerning the specification of *I* and *C* can be found in the electronic supplementary material.

Tree mortality occurs after continuous periods of growth depression [44]. Mangrove growth is therefore evaluated every year and trees die when the growth is less than 50% of the growth under optimal conditions for five consecutive years. The death of mangroves reduces the competition among the remaining trees, and therefore improves their growth conditions. Practically, when the product *I*⋅*C* in equation (2.3) is below 0.5 for five consecutive years, mangrove density decreases (reducing the total biomass which results in an increase in *C* in equation (2.3)) until the growth depression is halted and the product *I*⋅*C* thus equals or exceeds again a value of 0.5. In the case of no inundation stress (*I*=1), the self-thinning process (decrease in number of individuals) in the mangrove forest induced by competition among individuals eventually results in a mangrove density of 125 trees per ha. This maximum density of 125 mature trees per ha is obtained by applying the ‘zone of influence’ concept as described in detail in the electronic supplementary material. When inundation stress hinders the growth of mangroves (*I*<1), the final mangrove density is lower. All mangroves die and mangrove density reduces back to zero when inundation conditions become unfavourable for mangrove growth (*I*≤0.5). As inundation stress and bed elevation are coupled, the maximum density of mature trees is dependent on the bed level at which the trees are present. On the other hand, when conditions become more favourable for mangrove growth because of a decrease in inundation stress, mangrove density increases until the product *I*⋅*C* reaches a value of 0.5. The mangroves which are added to the model enter as young trees with a diameter of 1.37 cm, which is the initial stem diameter as mentioned above. As such, a single grid cell can contain mangrove trees of different sizes.

### (c) Effects of mangroves on physical processes

Mangroves influence hydrodynamics and sediment dynamics in various ways. The effects that we incorporated in the model are that mangroves:

— *Increase the drag coefficient* *C*_D_. Mangrove trees and their pneumatophores (aerial roots) offer additional resistance to the tidal flow and this effect is incorporated by defining *C*_D_ as a function of the projected area of mangrove vegetation and the volume of the vegetation. As a result, mangroves influence tidal flow velocities, and thus affect the magnitude of sediment transport.
— *Increase the erosion threshold* *θ*_cr_. Mangroves increase the sediment's resistance to erosion by the tidal flow as the roots of the mangroves play a role in stabilizing the sediments [48]. In our model, we therefore correlated *θ*_cr_ with the below-ground biomass. This results in reduced sediment transport rates.
— *Decrease slope-driven sediment transport* *S*_slope_. Because of the stabilizing character of the mangrove root system, *S*_slope_ decreases with increasing below-ground biomass.
— *Increase the bed elevation through organic matter production* Δ*Z*_org_. Mangroves produce organic matter and because the decomposition of the refractory component is extremely slow, organic matter builds up which can raise the soil surface by a few millimetres per year [49]. A recent study by Swales *et al*. [50] shows that the production of organic material also plays an important role in *A. marina* ecosystems. The main constituent of organic deposits in mangrove forests is usually refractory roots [51] and we thus related Δ*Z*_org_ to the below-ground biomass.

Further details regarding the parametrizations and implementation of these effects can be found in the electronic supplementary material.

### (d) Model set-up

**Figure 2.**
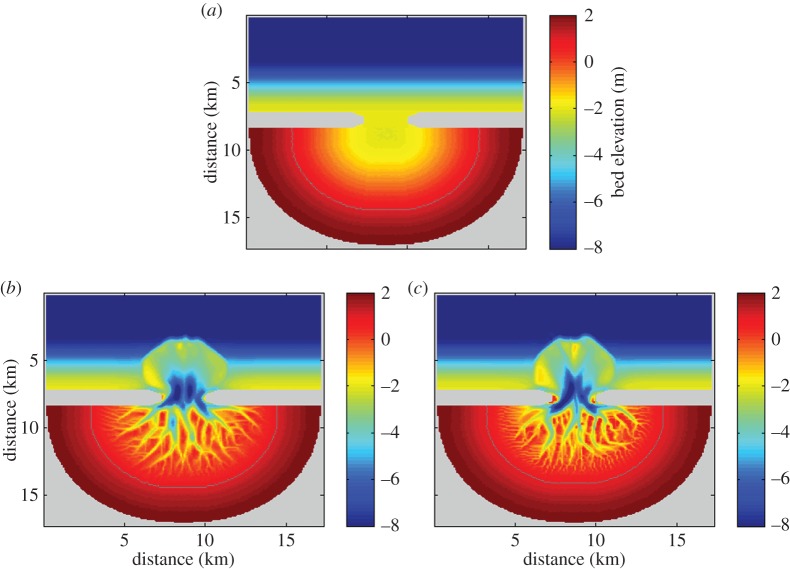
(*a*) Initial bathymetry used for the simulations and simulated morphology after 160 years (*b*) without and (*c*) with mangroves. Grey line represents the 1 m contour line which approximates the high-tide level.

An idealized and initially unchannelled bathymetry (figure 2*a*) was used for the numerical simulations performed in this study (this initial bathymetry is similar to the one used by van Maanen *et al.* [36,37]). It covers an area of 17×17 km and is composed of an offshore area, inlet and basin. Bed elevations in the offshore area increase from −8 m at the seaward boundary to −2 m at the entrance of the basin. The offshore area is separated from the tidal basin by the presence of land regions which formed impermeable and non-erodible barriers. Within the basin, bed elevations further increase towards +2 m at the landward boundary and random perturbations of −1.5 to +1.5 cm were added to the bed level in this area. The model is forced with a semidiurnal sinusoidal tide and the tidal range amounts to 2 m. The sediments are unimodal with a grain size diameter of 0.12 mm, representing very fine sand. A grid size of 100 m in both *x*- and *y*-direction is used.

Simulations previously reported have shown that the initial depth and the overall slope of the basin play a key role in the morphological evolution of the system [36]. The initial morphology adopted here is relatively shallow and in a non-equilibrium with hydrodynamic forcing conditions, resulting in the rapid formation of a tidal channel network. This has implications for mangrove establishment as the trees start growing in a dynamic environment, with mangrove colonization occurring first in the upper part of the basin, followed by the colonization of newly generated intertidal areas associated with the formation of the channel network.

### (e) Extracting the channel network

A key measure of the morphological evolution is the development of a channel network. To determine the density of channels, we extracted the channel network from the simulated morphologies using a technique based on the work by Passalacqua *et al.* [52]. This technique (previously used to analyse tidal networks [36,37]) includes nonlinear geometric filtering of the topography to enhance features that are critical to the network extraction. The geometric curvature of the isoheight contours is then calculated (see [26]). Channelized areas are characterized by positive curvature and channels are defined as areas where a sudden change in the statistical signature of the landscape occurs. We refer the reader to Passalacqua *et al*. [52] for further details.

## 3. Results

### (a) Morphological evolution with and without mangroves

The numerical model was first used to simulate the formation of a tidal channel network starting from the unchannelled initial bathymetry and in the absence of mangroves (figure 2*b*). This initial run showed that a fully developed channel network formed over long time scales, indicating that the interactions between hydrodynamics, sediment transport and the evolving topography are sufficient to give rise to channel pattern development. Large bathymetric changes occurred especially within the first few years as deep channels rapidly developed in the inlet of the tidal system. During ebb-tides, large volumes of sediment were transported towards the offshore area where decelerating flows caused sediment deposition and the formation of an ebb-tidal delta. In the tidal basin itself, channel initiation and the development of intertidal areas modified the morphology. Over time, as small channels grew larger and some of them branched, a complex network of channels emerged.

**Figure 3.**
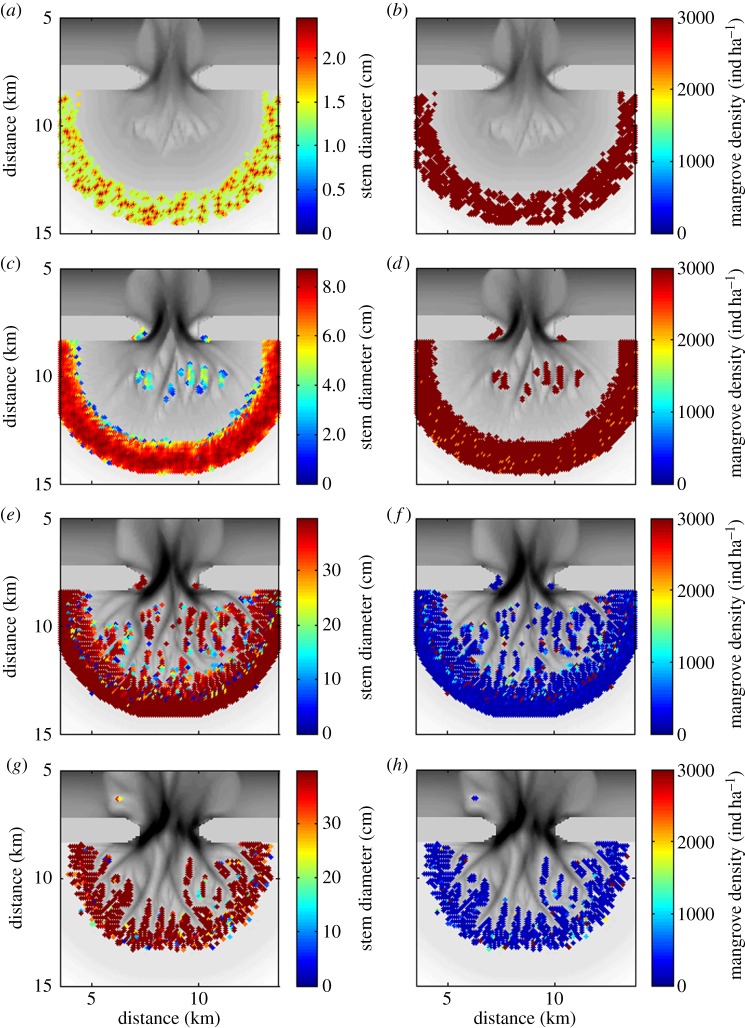
Stem diameter of the mangrove trees (in cm) after (*a*) 3, (*c*) 15, (*e*) 160 and (*g*) 1000 years of mangrove growth. Note that the subplots have a different colour-scale. Subplots (*b*,*d*,*f*,*h*) show the corresponding mangrove densities (in individuals per ha). Stem diameter of the trees and mangrove densities are superimposed on the bathymetry.

We used the initial model simulation without mangroves as a reference case against which to explore the effects of mangroves on morphological evolution. A second simulation was thus conducted, also starting from the unchannelled bathymetry, but in this case we allowed the growth of mangrove trees. Mangroves colonized bare areas when the inundation regime favoured mangrove establishment, and the trees started to have an effect on hydrodynamics and sediment transport processes as described in §2c. Figure 2*c* shows the simulated morphology after 160 years of mangrove growth, when mangroves had colonized nearly all areas above mid-tide. A detailed overview of mangrove growth is presented in figure 3. From figure 3*a*, which shows the diameter of the mangrove stems after 3 years, it can be easily detected where initial mangrove establishment took place (and from where mangroves expanded to neighbouring grid cells), as this is where the mangrove trees are the largest. During the first 15 years of mangrove growth, the trees were still relatively small (figure 3*c*) so that the limited competition among trees allowed for the presence of 3000 trees per ha (figure 3*b*,*d*), which equals the initial density. While the trees become larger in size, total biomass is increasing which increases the tree-to-tree competition. This in turn hinders the growth of individual trees (as *C* in equation (2.3) decreases) which ultimately drives tree mortality and a reduction in mangrove density. The majority of mangrove trees had a stem diameter of more than 35 cm after 160 years (figure 3*e*) and for these trees the mangrove density was around 125 individuals per ha (figure 3*f*).

**Figure 4.**
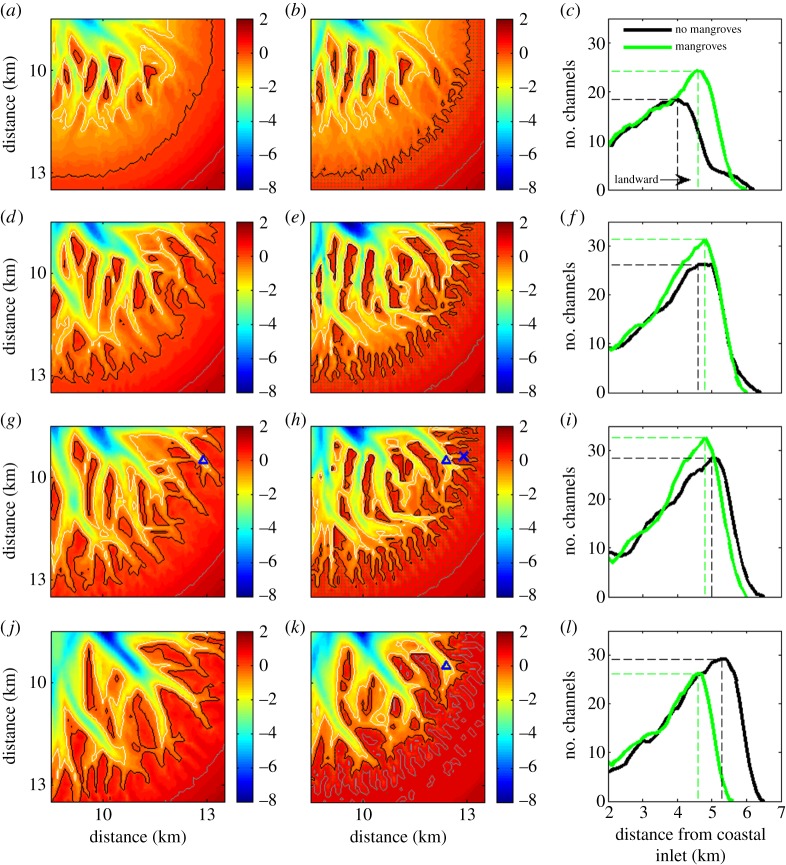
Simulated morphologies after 20, 80, 160 and 1000 years (*a*,*d*,*g*,*j*) without and (*b*,*e*,*h*,*k*) with mangroves. White, black and grey lines represent −1 (low-tide), 0 (mid-tide) and 1 (high-tide) m contour lines, respectively. The dots on panel (*b*,*e*,*h*,*k*) indicate the grid cells where mangroves are present. Plots with number of channels versus distance from the coastal inlet for the unvegetated (black) and vegetated (green) morphologies are shown in (*c*,*f*,*i*,*l*). The number of channels is shown as a moving average over 500 m (five grid cells). The dashed lines in these plots indicate the maximum number of channels and associated location. The blue triangles in (*g*,*h*,*k*) and blue cross in (*h*) indicate the locations for which the stage–velocity curves, as shown in figure 7, are developed.

Important differences can be observed when the evolution of the channel network in the unvegetated and vegetated scenario is compared in detail (figure 4). The number of channels as a function of distance from the inlet is plotted in the third column of figure 4 and shows that there is a general initial increase in the landward direction, because of the naturally branching character of the network. Channel occurrence then declines as their landward extent is halted. Figure 4*c* shows that mangroves enhance the initial formation of channels as it can be observed that the channel network has advanced further in comparison to the unvegetated scenario. This is particularly evident when the 0 m contour lines of both bathymetries are followed (black lines in figure 4*a*,*b*). The 0 m contour line refers to the elevation above which mangrove trees occur and in the presence of mangroves this contour line has become somewhat irregular after 20 years, which is related to erosion and deposition as a result of the formation of channels.

After the initial stage of channel formation, the headward erosion and branching of channels in subsequent decades drives expansion of both networks (with and without mangroves), although the rate at which the channel network expands in the presence of mangroves diminishes over time. More specifically, from 20 to 80 years, morphological evolution causes channel occurrence to increase significantly (compare green lines in figure 4*c*,*f*), while in the following period towards 160 years the network remains relatively static with only small changes in channel distribution (figure 4*f*,*i*). After 160 years, the effects of mangroves on hydro- and sediment dynamics have contributed to the development of a dense channel network (figure 4*h*). However, it is the unvegetated basin in which the channels have extended landward the furthest (figure 4*i*; black line exceeds green line in upper part of basin).

When assessing the channel network evolution over even longer time scales, it can be seen that, while the unvegetated network continues to expand, a strong reduction in channel occurrence takes place in the vegetated scenario (figure 4*l*). This process is related to organic matter production. That is, in addition to the transport of inorganic sediments, organic matter accumulation in areas covered by mangroves causes the bed elevation to increase up to a few millimetres per year. Vegetated areas in the upper part of the tidal basin therefore continue to raise their bed elevation until the soil surface reaches a level at which it is no longer inundated. At that point, mangrove growth is reduced by 50% (see electronic supplementary material, figure S1) and tree mortality follows, such that the production of organic matter ceases as well. The loss of mangroves in the upper region of the basin can also be seen in figure 3*g*,*h*. This die-back of mangroves in those areas that become only rarely inundated has been described and observed for natural mangrove systems [53]. An important related effect is that the high-tide mark consistently moves seaward (grey line in figure 4*k*), reducing the total surface area that gets flooded during a tidal cycle. This in turn affects the tidal prism. In fact, not only the total water volume entering the basin through the inlet is reduced as a consequence of a smaller total inundated area, but also the discharge of the channels in the upper basin is affected. As will be shown in §4b, this results in weaker tidal flows, ultimately causing channel infilling. It is this infilling of channels, together with the ongoing accretion in vegetated areas, that causes the seaward retreat of the tidal channel network as indicated in figure 4*l*.

**Figure 5.**
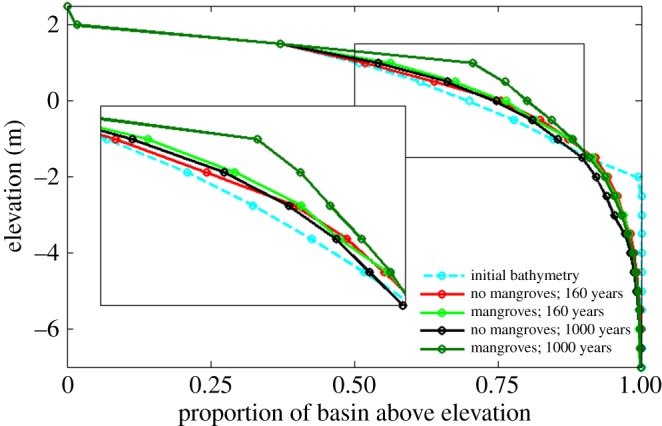
Hypsometric curves for the simulated morphologies with and without mangroves after 160 and 1000 years.

The production of organic matter also plays a role in altering the overall hypsometry of the tidal basin. Figure 5 shows hypsometric curves for the initial bathymetry, as well as for the unvegetated and vegetated morphologies at different moments in time. These curves can be used to characterize large-scale bathymetric changes [36,54]. Initially, sediment is mainly redistributed from the deep areas to the shallow areas, such that more intertidal areas are generated. The hypsometric curves of the unvegetated and vegetated morphologies after 160 years (compare red and light shaded green lines in figure 5) indicate that, although the differences are small, a larger proportion of the bed elevations occur within the upper portion of the intertidal zone when mangroves are present. From these curves, it is difficult to tease apart the relative contributions of inorganic and organic accretion in causing this difference. However, the clear change in hypsometry for the vegetated morphology after 1000 years (dark shaded green line in figure 5), whereby a considerably larger area occurs above a bed elevation of 1 m is inevitably driven by the ongoing production of organic matter. By then, the hypsometric curve has flattened for the higher elevations and exhibits a shape which deviates from the typical sigmoidal shape as observed for the simulated morphologies without mangroves and described by, for example, Boon & Byrne [54]. This effect becomes dominant only over longer time scales as the accretion of organic material is a slow but gradual process [49].

### (b) Effects of mangroves on hydrodynamics

**Figure 6.**
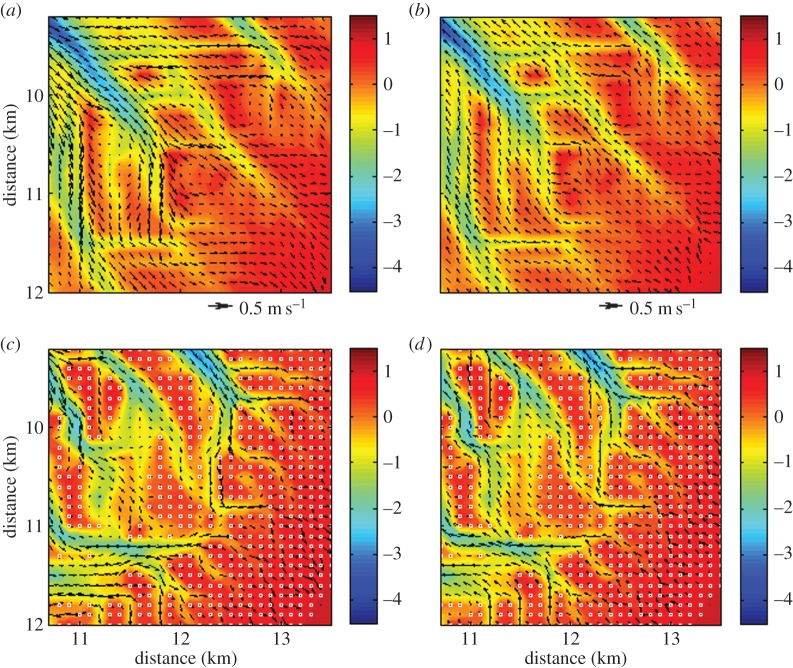
Flow field during (*a*,*c*) rising and (*b*,*d*) falling tide over a part of the (*a*,*b*) unvegetated and (*c*,*d*) vegetated morphologies after 160 years. Arrows on the panels represent magnitude and direction of the flow. Scale of the arrows is indicated in between the panels. The white circles on panel (*c*,*d*) indicate the grid cells where mangroves are present.

The differences in morphological evolution as described above are partly driven by the way mangroves alter the characteristics of the tidal flow. Therefore, with the purpose of unravelling the mechanisms with which mangroves affect channel network dynamics, we extracted the flow fields during rising and falling tide over both the unvegetated and vegetated morphologies. Figure 6 shows that the direction as well as the magnitude of the flow is affected by the presence of mangrove trees. During rising tide, the flow in the vegetated scenario (figure 6*c*) is mainly confined within the tidal channels. When areas with mangroves become inundated, the extra drag caused by the trees and pneumatophores drives a strong reduction in the magnitude of the flow. In terms of sediment dynamics, this leads to negative gradients in sediment transport fluxes and thus an increase in deposition and bed elevation. On the other hand, the extra flow resistance in mangrove forests results in flow concentration and sediment erosion in between vegetated areas, enhancing thus the formation of channels at those locations. When mangroves are absent, the flow is less constricted to the channel network (figure 6*a*). The flow over intertidal areas also reaches higher velocities in comparison with the vegetated scenario.

Similar differences in the flow characteristics can be observed between the vegetated and unvegetated scenarios during falling tide (figure 6*b*,*d*). In the absence of mangroves (figure 6*b*), the water is drained through the channels as well as over the intertidal areas. The magnitude of the flow is relatively homogeneous and the flow is primarily directed towards the main inlet of the tidal basin (the left-top corner in figure 6*b*). By contrast, the flow field is rather heterogeneous in the vegetated scenario with smaller velocities occurring within the mangrove forest (figure 6*d*). The flow is often directed towards the nearest channel through which the water is then subsequently drained. The tidal flow thus concentrates again within the channels. As will be discussed in more detail in §4a, similar types of flow patterns and feedbacks have been described for salt marsh systems [30,55].

**Figure 7.**
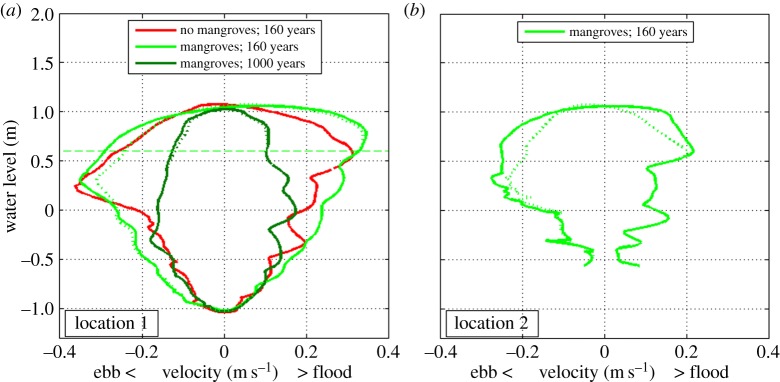
Stage–velocity curves for channels at two different locations in the presence and absence of mangroves. Location 1 corresponds to the triangles in figure 4*g*,*h*,*k*. Location 2 corresponds to the cross in figure 4*h*. The dotted lines correspond to stage–velocity curves for the morphologies with mangroves, but for which the drag coefficients have been reset to the unvegetated value. The horizontal dashed line in (*a*) indicates the channel bank elevation of the channel flanked by mangroves after 160 years.

Stage–velocity curves provide an additional way to study flow characteristics in tidal environments [56,57]. We therefore developed several such curves for channels in the simulated morphologies with and without mangroves (figure 7). Focusing first on the flow in the unvegetated basin, we can observe that the stage–velocity curve for a particular channel (red line in figure 7*a*) reveals the typical asymmetry in flow between flood and ebb [58–60]. Fagherazzi *et al.* [27,56] discussed this asymmetry in detail and they describe the occurrence of two distinct surges in the tidal flow, one when the adjacent intertidal platform is being flooded and one when the platform has drained. They also point out that it takes time for the water to flood and drain the platform such that the maximum ebb and flood currents occur at different water levels. Although these concepts have been described in the context of salt marshes for which the intertidal platform is nearly flat, a similar type of asymmetry in the tidal flow exists for the simulations here as the maximum ebb flow occurs at an elevation of 0.25 m, while the maximum velocity during flood occurs at an elevation of 0.57 m (figure 7*a*).

Examining the stage–velocity curve for a channel flanked by mangroves (light shaded green line in figure 7*a*) further highlights their effects on the tidal flow. An obvious difference is the higher water elevation at which the maximum flood velocity is reached (elevation equals 0.78 m). This is related to the channel banks, which for this channel have become higher in the presence of mangroves. In fact, the channel banks of this channel are at an elevation of approximately 0.6 m (which is higher than the water elevation at which the flood surge is occurring when mangroves are absent, as described above). As expected, the increase in channel bank height causes the flood surge to occur later within the tidal cycle. An additional difference caused by mangroves can be found in the overall strength of the tidal current. When mangroves are present, the magnitude of the flow velocity is higher for nearly the entire tidal cycle. Over longer time scales, the reduction in tidal prism feeds back into the dynamics of the channels promoting other longer term changes in the overall character of the landscape. The stage–velocity curve after 1000 years (dark shaded green line in figure 7*a*) shows that the tidal current has become significantly weaker over time, with flow velocities not exceeding 0.2 m s^−1^, neither during ebb or flood.

Mangroves influence stage–velocity curves by affecting the morphological evolution (such as changing the height of channel banks), as well as by increasing flow resistance. To separate these effects, we extracted the curves for the vegetated morphologies with drag coefficients reset to the standard value indicative of no mangroves. For the stage–velocity curves discussed above, leaving out the drag only has a minor effect (compare solid and dashed green lines in figure 7*a*). However, for a channel located further within the mangrove forest (see blue cross in figure 4*h* for location), the influence of mangroves on drag does have an important effect on the way the tidal current propagates through the channel (compare solid and dashed green lines in figure 7*b*). Neglecting the extra flow resistance results in smaller flow velocities, especially for the higher water levels. This again indicates that the increased drag by mangroves enhances the flow within the channels.

### (c) Effects of mangroves on channel density

As described in §2c, mangroves affect hydrodynamics and sediment dynamics by increasing both the drag coefficient and the erosion threshold, decreasing the slope-driven sediment transport and by producing organic matter. Differences in the channel networks of the unvegetated (figure 2*b*) and vegetated (figure 2*c*) morphologies are the cumulative result of these effects. To enhance our understanding of how mangroves affect the formation of tidal channels, we performed additional simulations to elucidate how each of these single effects that mangroves have on physical processes influence channel formation.

**Figure 8.**
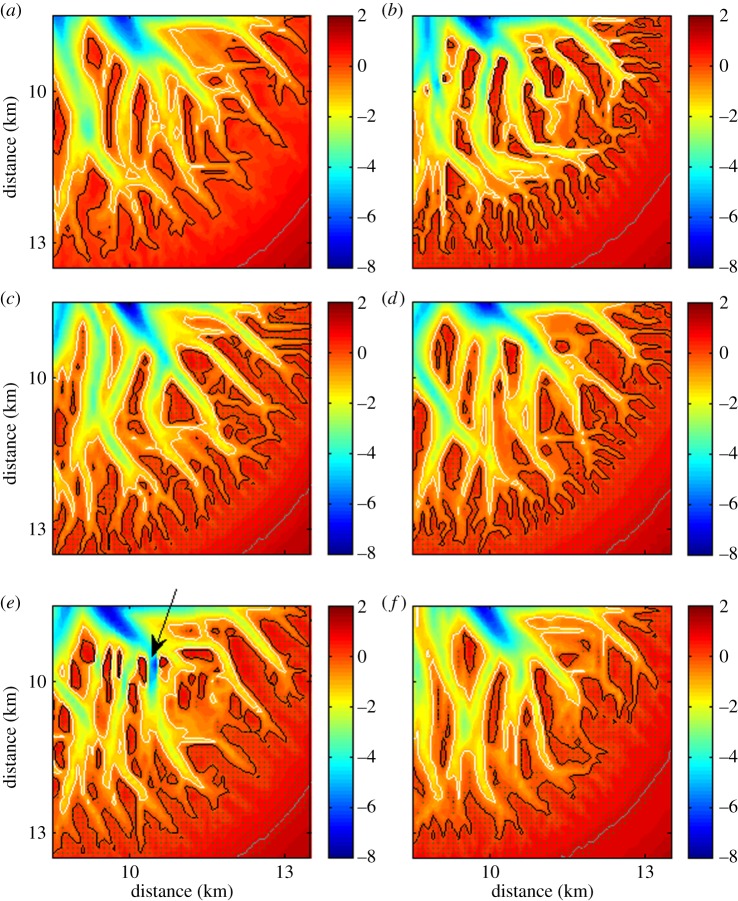
Simulated morphologies after 160 years (*a*) without mangroves, (*b*) with mangroves, and when only the effect of mangroves on (*c*) *C*_D_, (*d*) *θ*_cr_, (*e*) *S*_slope_ and (*f*) Δ*Z*_org_ was included. White, black and grey lines represent −1 (low-tide), 0 (mid-tide), and 1 (high-tide) m contour lines, respectively. The arrow in (*e*) indicates a deep channel with steep banks. The dots indicate the grid cells where mangroves are present.

**Figure 9.**
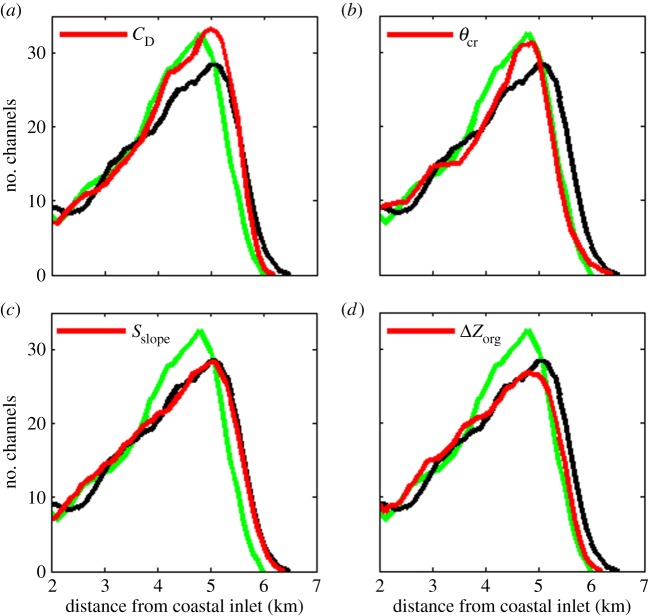
Number of channels versus distance from the coastal inlet for the unvegetated (black) and vegetated (red) morphologies after 160 years. For the vegetated scenarios, only the effect of mangroves on (*a*) *C*_D_, (*b*) *θ*_cr_, (*c*) *S*_slope_ and (*d*) Δ*Z*_org_ was included. The number of channels is shown as a moving average over 500 m (five grid cells). The green lines indicate the number of channels when all the four effects are taken into account (similar to the green line in figure 4*i*).

Four additional simulations were performed and each time only one out of the four effects of mangroves was included while the remaining three were ignored. Figure 8*c*–*e*,*f* shows part of the simulated morphology after 160 years, including the effect of mangroves on, respectively, *C*_D_, *θ*_cr_, *S*_slope_ and Δ*Z*_org_. Due to the extra drag produced by the trees and pneumatophores, mangroves obstruct the flow which leads to flow convergence and increased erosion in the areas between vegetated regions. As a result, channel formation is enhanced in the simulation used to assess the importance of the extra flow resistance in areas covered by mangroves (cf. figure 8*a*,*c*). The maximum number of channels at a specific distance from the coastal inlet is slightly higher when only the effect of mangroves on drag is included than when all four effects are taken into account (compare red and green line in figure 9*a*). Moreover, in the upper part of the basin (where channel occurrence is decreasing again), channel density in the unvegetated and vegetated scenario with only drag included is nearly equal. This suggests that the extra drag force caused by mangroves is not the main factor responsible for the limited ability of tidal channels to expand landward in vegetated basins (as was noted when discussing figure 4*i*; see also green line in figure 9*a*).

The additional simulation to investigate how a changing erosion threshold influences morphological evolution shows that its effect on channel formation is twofold. Incorporating a critical mobility parameter, which increases within the mangrove forest, results in a strong reduction in sediment transport fluxes once the flow encounters mangroves trees. Net sediment deposition in the mangrove forest then allows the difference in bed elevation between the unvegetated channel and the adjacent vegetated platform to increase, giving rise to a positive feedback. Over longer time scales, the vegetation-induced variation in the sediment's resistance to erosion drives additional branching of the channels, increasing channel density (figures 8*d* and 9*b*). At the same time, mangroves which cover the area at the headward side of the channels decrease the erodability of the sediment and hinder the possibility of these channels to expand further landward. Consequentially, the number of channels starts to decrease relatively close to the coastal inlet in comparison to the unvegetated morphology, resulting in a lower channel density in the upper part of the basin (compare red and black lines in figure 9*b*).

Channel density remains essentially unaltered when only the effect of mangroves on slope-driven sediment transport is included (figure 9*c*). However, the strength of channel banks increases when they are covered with mangroves because of the stabilizing character of the roots. As such, mangroves affect the cross-sectional shape of channels because the increase in bank stability allows the channels to develop steeper banks (see the arrow in figure 8*e*).

Not surprisingly, the expansion of the channel network is hindered in the simulation for which only the production of organic matter is included. As highlighted earlier, the vertical growth of the vegetated areas reduces the volume of water stored on these vegetated platforms, in turn reducing the tidal prism flowing through the tidal channels. As a result, current velocities and the erosive power of the channels decrease as well. This has large-scale implications as the channels do not have the ability to extend landward as far as in the unvegetated scenario and, after 160 years, a less dense channel network in the higher regions of the embayment can be observed (figures 8*f* and 9*d*).

### (d) Morphological evolution under a rising sea level

**Figure 10.**
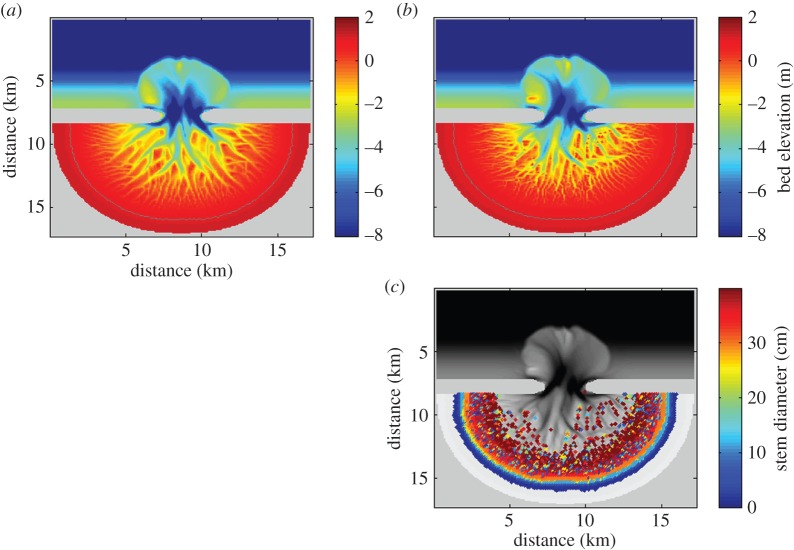
Simulated morphology after 160 years of sea-level rise at a rate of 5.0 mm yr^−1^ (*a*) without and (*b*) with mangroves. Sea level started to rise after 160 years of morphological evolution under a stable mean water level (the morphologies shown in figure 2). Grey line represents the 1 m contour line which approximates the high-tide level. (*c*) Stem diameter of the mangrove trees (in cm), showing the landward migration of mangroves.

**Figure 11.**
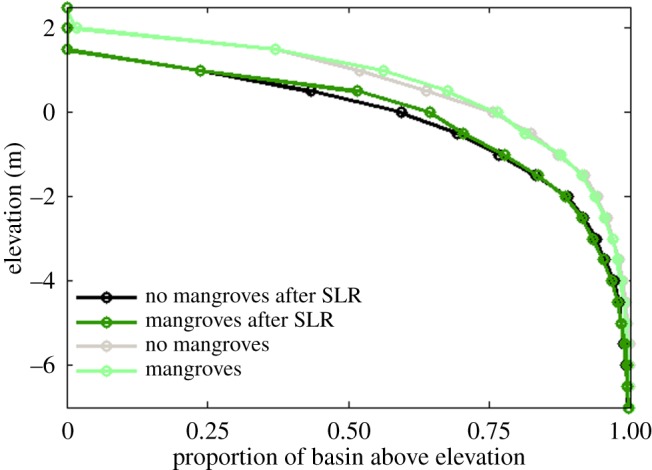
Hypsometric curves for the simulated morphologies with and without mangroves before (figure 2*b*,*c*) and after (figure 10*a*,*b*) sea-level rise.

Sea-level rise has a strong impact on tidal embayments and the morphological response of these systems to sea-level rise may be significant. To explore the effects of mangroves on possible changes in the morphology and characteristics of the channel network, we subjected the landscapes shown in figure 2 to an increase in sea level. The rate of sea-level rise for these particular simulations was set to 5.0 mm yr^−1^. In the vegetated basin, some of the shallow areas close to the inlet, although significantly reduced in size, still have a bed elevation above 0 m after 160 years, which is sufficient for the growth of mangroves (figure 10*b*,*c*). The hypsometric curves of the basins after sea-level rise (figure 11) indicate that the proportion of bed elevations above 0 m is around 5% larger when mangroves are present and around 8% larger with respect to the 0.5 m bed elevation. Mangroves and the related soil expansion driven by organic matter production can thus potentially play an important role in enhancing the ability of the soil surface to maintain an elevation within the upper portion of the intertidal zone while the sea level is rising.

**Figure 12.**
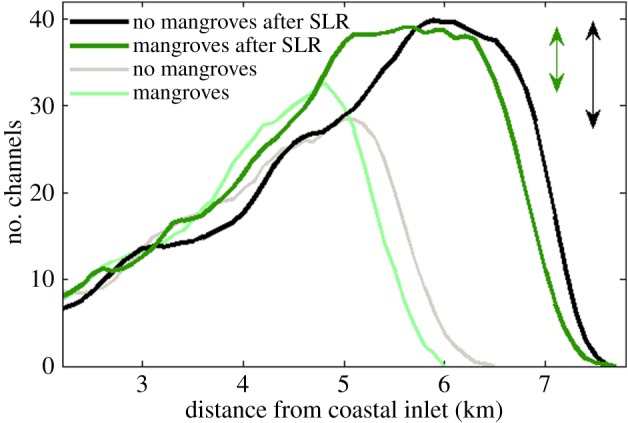
Number of channels versus distance from the coastal inlet for the unvegetated and vegetated morphologies before and after sea-level rise. The number of channels is shown as a moving average over 500 m (five grid cells). The arrows indicate the increase in the maximum number of channels due to sea-level rise.

Sea-level rise induces a decrease in channel density for a small part of the basin (for the unvegetated basin, this is between 3.0 and 4.4 km; compare grey and black lines in figure 12). This is caused by the increase in water depth which drives the channels to become larger and more widely spaced (see also [37]). In addition, sea-level rise results in headward erosion of the channels and expansion of the overall network, a process which is influenced by the presence of mangroves. Where the channels expand landward, the number of channels increases more substantially in the unvegetated basin than in the basin with mangroves (as highlighted by the arrows in figure 12). This indicates that the branching of channels is hindered when the channels expand into an area which is completely covered with mangroves. The limited ability of the channels to branch while expanding landward in the vegetated basin is further indicated by the channel occurrence which remains nearly constant between 5 and 6 km from the inlet. A nearly constant channel occurrence implies that the channels are extending landward, but without being subject to a regular branching process. After sea-level rise, the existence of channels near high tide is still suppressed when mangroves are occupying the basin, partly related to the difficulty for channels to carve through vegetated surfaces.

## 4. Discussion

### (a) Influence of vegetation on channel network formation and evolution

Elements of our simulations reflect previous studies that have focused on tidal channel networks that are influenced by salt marsh vegetation. Temmerman *et al*. [30], for example, applied a morphodynamic model which accounted for the effects of salt marsh plants on drag and turbulence to simulate channel formation on an initially bare tidal flat. Model results showed that channel drainage density increases with denser vegetation and it was concluded that the flow concentration between vegetated patches is responsible for the enhanced channel erosion. Over recent years, these biophysical interactions in salt marsh settings have received particular attention [61–63]. Our simulations of large-scale channel network-forming processes indicate that mangroves give rise to similar types of feedbacks. The additional flow resistance in mangrove forests has shown to drive enhanced flow concentration and thus erosion, giving rise to a denser channel network.

The effect of vegetation on increasing the erosion threshold and the influence of this on channel formation has received less attention. Here, we have shown, however, that a spatial variation in the sediment's resistance to erosion, governed by the presence of vegetation, contributes to the additional branching of the channels. An increase in the erosion threshold where mangrove trees are present leads to extra sedimentation in the forest. This indicates that, in addition to the vegetation-induced channel erosion, enhanced forest deposition also plays an important role in the formation of the channel network. While these ecogeomorphological feedbacks can enhance channel initiation and branching, our model results indicate that the branching of channels is hindered when sea level is rising. Sea-level rise causes the channels to expand landward, but when this expansion occurs over a surface which is completely covered with mangroves, the feedback mechanisms leading to, for example, enhanced flow concentration do not operate as effectively. Channel development is thus accelerated in areas which are only partly vegetated.

The observed seaward retreat of the channel network over longer time scales occurs through a combination of organic accumulation, reduction in tidal prism, and channel infilling. This is consistent with model results described by D'Alpaos *et al.* [64], who simulated the cross-sectional evolution of a salt marsh channel and showed that infilling of the channel can indeed occur after the emergence of a marsh platform. Contrasting responses were reported by Vandenbruwaene *et al*. [62] who analysed a series of aerial photographs and digital elevation models to study how the geometric properties of tidal channels changed while the intertidal marsh platform accreted. They found that continued sediment accretion and tidal prism reduction did not affect channel drainage density or channel dimensions (although their study did not explicitly assess the effect on channel depth and instead focused on channel width). It was hypothesized that the smaller tidal prism only reduced the sheet flow over the platform, while the flow in the channel was not much affected. Clearly, whether a reduction in tidal prism affects the landscape-forming flow is dependent on the geomorphic setting and on how vegetation alters flow routing patterns; a topic which deserves further investigation.

The changes in tidal prism noted above may provide insight into the process of mangrove establishment. For example, do mangroves dictate morphological evolution and create/modify their own habitat by actively building land, or are mangroves more passive and do they respond to depositional processes rather that causing them [65–68]? Although our simulations only concern idealized cases, they indicate that infilling of the channels leads to increased channel bed elevations and can ultimately generate a bed surface which is sufficiently high to support the growth of mangrove trees. In this way, mangroves modify the physical environment by reducing channel depths and can potentially play a role in creating their own habitat. In this context, it should be emphasized that the morphologies presented here after 1000 years were still evolving under the influence of mangroves. It is difficult to anticipate whether ongoing sedimentation and accretion would ultimately result in complete infilling of the simulated basin as these processes occur over much longer time scales (see also [69]).

### (b) Modelling approach, limitations and perspectives

The simulations presented here provide insight into the effects of *A. marina* mangroves on channel network evolution. A number of assumptions, however, were made throughout model development and included processes were simplified. The parametrizations used here to describe how mangroves affect physical processes and vice versa were based on previous studies performed by other authors combined with common and widely accepted knowledge of the system. While these parametrizations capture the main processes governing the dynamics of mangrove environments in natural systems, some other features of vegetation dynamics are neglected. The growth of mangroves, for example, is a complex process and tree growth may be constrained by salinity stress, availability of nutrients and temperature. To keep the numerical model relatively simple and model output as transparent as possible, we preferred to include only a minimum number of processes. We decided to limit the growth of the mangrove trees only by inundation and competition stress. Incorporating just these two stresses allow the numerical model to reproduce important behaviour typical of mangrove forests: the self-thinning process due to the competition among trees and the limited growth of the trees when they are inundated for either longer or shorter than the optimum inundation period.

The sediment transport module applied in this study is based on the Engelund and Hansen formulation [40]. Although this approach has been widely used to simulate the long-term evolution of tidal systems [70,71], there are certain limitations associated with the formulation that should be considered. For example, it only allows modelling of non-cohesive sediments and it does not distinguish between bed load and suspended load. This has implications for the implementation of the biophysical interactions. In the ecomorphodynamic model presented here, mangroves modify sediment transport rates directly by increasing the erosion threshold and indirectly by reducing the strength of the tidal currents in the forest. Other effects such as enhanced settling and the direct capture of sediment particles on the tree stems and pneumatophores are not included as these processes apply more to the dynamics of cohesive sediments [72], while our study focuses on sandy environments. Also, to parametrize the slope-driven sediment transport term, we used the formulation described by Kirwan & Murray [31]. While they adopted this formulation for salt marsh channels, it should be noted that the approach on which this formulation is based was initially developed for fluvial channels [73]. Further research is clearly needed to test if these approaches are transferable and to develop more accurate representations of gravitationally driven sediment fluxes. In terms of hydrodynamics, we included the effect of the trees and pneumatophores by increasing the drag coefficient. While this is sufficient to capture important feedbacks occurring in mangrove ecosystems, other vegetation effects such as modifying the turbulent field [74] are not included.

Another example of a complex process which is difficult to capture in numerical models is the accumulation of organic material. Organic accumulation is the result of the balance between the production and decomposition of organic matter, and field measurements indicate that this balance varies widely across different settings [49]. Field data as described by McKee *et al.* [75] revealed a tight coupling between the organic accumulation of Caribbean mangroves and sea-level rise. This finding was suggested to be the result of flooding effects on the balance between subsurface root production and decomposition. Despite the complexity of the process, we followed a relatively simple (but commonly used) approach and linearly related soil expansion to the below-ground biomass. In addition, the model used here was developed to capture the dynamics of sandy shoals colonized by mangroves. This implies that the accumulation of organic material altered the bed composition, but this effect was not included in the model.

Linking the types of model results presented here to field observations is not straightforward, especially given the large spatial and temporal scales involved. A potential way forward lies in the use of remote sensing. Aerial photographs have already been used in the study of tidal channel dynamics in salt marsh systems [30,62,63]. As the availability of such images is continuously increasing, as well as the time span over which they are obtained, this opens up extra possibilities to analyse how mangroves affect channel network formation. At the same time, such remote sensing-based analyses would provide ways to further test our model results. In the electronic supplementary material, we have included aerial photographs that show how the geometry of a river flowing into a New Zealand estuary was subject to several changes while mangrove growth expanded. Remote sensing studies of dynamic mangrove settings elsewhere, together with the development of new methodologies that allow for a detailed analysis of channel network characteristics (e.g. [76]), can help to close the gap between numerical modelling and field observations.

The model presented here dynamically couples biotic and abiotic processes and is used to simulate the large-scale and long-term morphodynamics of tidal basins. Future model development could potentially help to address a wide range of environmentally inclined questions. Ecomorphodynamic model simulations could for example shed light on the consequences of the removal of mangrove trees for morphological change and the overall system's functioning. This is relevant as mangrove forests are in rapid decline as they are cleared for aquaculture, urbanization or coastal landfill [1]. Also, the ability of mangrove forests to continue providing important ecosystem services (such as carbon sequestration) is dependent on how the geomorphic system and the ecosystem itself responds to climate change impacts, including sea-level rise. Models that account for the complex biophysical interactions are needed to improve our understanding of such issues which inevitably require an interdisciplinary approach.

## 5. Conclusion

We developed an ecomorphodynamic model to explore the effects of *A. marina* mangroves on the long-term evolution of sandy tidal embayments. The model accounts for the effects of mangroves on hydrodynamics and sediment dynamics. In turn, hydrodynamic conditions affect the colonization, growth and mortality of the trees so that a two-way coupling between physics and biology arises. Parametrizing such biophysical feedbacks is not straightforward and the selected interactions have been incorporated here based on previous field and analytical studies, as well as other modelling efforts. Simulations were carried out starting from an unchannelled tidal basin and results indicate that mangroves have a strong control on tidal channel network evolution. The enhanced branching of tidal channels in the presence of mangroves, which results in a higher channel density, is mainly caused by the additional drag produced by the trees and its pneumatophores. The extra flow resistance in mangrove forests drives flow concentration and sediment erosion in between vegetated areas and thus enhances the formation of channels. The increase in the erosion threshold when the surface is covered with mangroves has contrasting effects. The process of channel branching is again enhanced. On the other hand, headward erosion of the channels is reduced so that channel density decreases in the upper part of the tidal embayment. The accumulation of organic matter leads to a reduction in the tidal prism which flows through the channels, reducing the extent to which the channels are expanding landward and ultimately causing a seaward retreat in the overall network. The decrease in slope-driven sediment transport in areas with mangroves allows the channel banks to become steeper, but does not have an effect on channel density. Simulated morphological evolution under a rising mean water level indicates that channel networks expand landward during sea-level rise. However, when channels expand into an area covered with mangroves, channel formation is hindered which decreases both the branching and headward erosion of channels. Overall, the model simulations presented here highlight the role of mangroves in the morphological evolution of tidal embayments and further emphasize the need to include biophysical interactions in morphodynamic models.

## Supplementary Material

Supplementary material

## Data accessibility

The hydrodynamic solver used in this research has been obtained from the Centre for Water Research (University of Western Australia): http://www.cwr.uwa.edu.au/software1/. The software to extract the channel networks from simulated morphologies can be found on: https://sites.google.com/site/geonethome/source-code. The additional information supporting this article has been uploaded as part of the electronic supplementary material.
