# Supplementary material for: On the ecogeomorphological feedbacks that control tidal channel network evolution in a sandy mangrove setting
Source: Proc Math Phys Eng Sci. 2015 Aug 8;471(2180):20150115. doi: 10.1098/rspa.2015.0115 (PMC4550008; doi:10.1098/rspa.2015.0115)
Supplement: Supplementary material [file rspa20150115supp1.pdf]

Supplementary material for

On the ecogeomorphological feedbacks that control tidal channel network evolution in a sandy mangrove setting

B. van Maanen, G. Coco and K.R. Bryan

1. Specification of inundation and competition stress factors

When developing the correction factors for mangrove growth resulting from inundation (I) and competition (C) stress, it is of specific interest when these factors reach a value of 0.5, as this determines when mangrove mortality occurs. I is a function of the hydroperiod and we relate the two as:

$$I = a \cdot P + b \cdot P^2 + c\,, \qquad (1.1)$$

where P represents the relative hydroperiod ($T_{(inundated)}/T_{(tide)}$) and a, b, and c are constants which have been set to 4, -8, and 0.5, respectively. Equation (1.1) suggests that there is a maximum growth rate for a specific hydroperiod. Growth rates are reduced when the mangroves are inundated for either longer or shorter (figure S1a). The values of a, b , and c were chosen such that I = 0.5 when the mangroves are inundated for half of the time (P = 0.5) or when the mangroves are not inundated at all (P = 0). Mangrove trees stop growing completely when they are inundated for longer periods of time (P > 0.6).

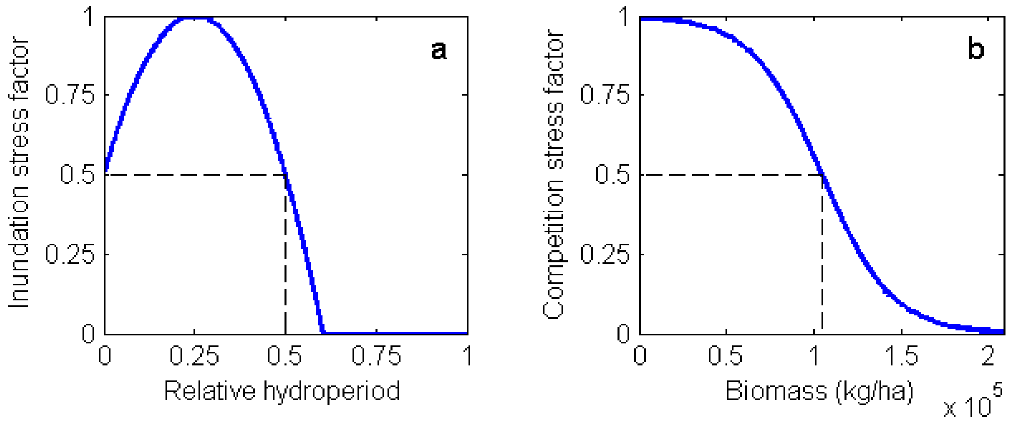

Figure S1. The correction factors for mangrove growth resulting from (a) inundation and (b) competition stress. A relative hydroperiod of 1 indicates permanent inundation. The dashed lines indicate where the stress multipliers reach a value of 0.5.

C is determined by the total biomass B and we apply a sigmoid function to relate the two (figure S1b):

$$C = \frac{1}{1 + \exp\left[d\left(B_{0.5} - B\right)\right]}, \quad (1.2)$$

where d is another constant and is set to -0.00005 and $B_{0.5}$ is the value of B for which $C = 0.5$. In order to compute B, we consider the total number of mangrove trees per grid cell and the weight of each single tree W_{tree}, which is given by the summation of the above-ground $W_{tree,a}$ and below-ground $W_{tree,b}$ tree weight:

$$W_{tree} = W_{tree,a} + W_{tree,b}. \quad (1.3)$$

The above-ground and below-ground tree weight of the *Avicennia marina* is given by the following allometric relationships [1]:

$$W_{tree,a} = 0.308 D^{2.11} \text{ and} \quad (1.4)$$

$$W_{tree,b} = 1.28 D^{1.17}, \quad (1.5)$$

where D is the stem diameter (cm). To get an estimate of the maximum number of trees that can be present in a single grid cell, we used the concept of the so-called 'zone of influence'. *Berger and Hildenbrandt* [2] applied this approach by considering a circular zone around each tree in which the tree exploits resources. They proposed a simple relationship to evaluate the radius R of this zone:

$$R = 10\sqrt{0.5\frac{D}{100}}. \quad (1.6)$$

Application of equation (1.6) leads to the outcome that 125 mature (D=D_{max}=40 cm) mangrove trees can be present per ha (size of a grid cell) without mangroves having to compete for resources (the condition in which the circular zones around trees used for resources are not overlapping). As such, $B_{0.5}$ in equation (1.2) is set to $1.04 \cdot 10^5$ kg/ha which is the total biomass associated with 125 mature trees. *Berger and Hildenbrandt* [2] applied

the ‘zone of influence’ concept within an individual-based model which evaluates the growth of each single tree. Clearly, the dynamics that involve individual trees are beyond the scale of interest for the model developed in this study. The ‘zone of influence’ used in this context should only be seen as a way to obtain an estimate of the maximum number of mangroves in a grid cell.

2. Implementation of the effects of mangroves on physical processes

Aquatic plants are well-known for offering additional resistance to the flow [3]. Mazda et al. [4,5] performed a theoretical analysis based on the momentum equation to show that the drag force by mangroves is strongly dependent on the total projected area of obstacles A and the total volume of obstacles V_M in a certain control volume V. They related the drag coefficient to a characteristic vegetation length scale L which was defined as:

$$L = (V - V_M)/A. \quad (2.1)$$

The height of control volume V equals the local water depth and L can thus vary during a tidal cycle [4,5]. In addition, L contains information about the spacing between the trunks and roots of the mangrove trees and L decreases (decreasing spacing) with increasing V_M and A. The drag coefficient C_D is thus inversely correlated with L and the effect is here described by:

$$C_D = C_{D,no} + eL^{-1}, \quad (2.2)$$

where $C_{D,no}$ is the drag coefficient when no mangroves are present and this is set to a standard value of 0.005. e is a dimensional constant and set to 5 m to obtain realistic values for C_D. The application of equation (2.2) requires a description of the number of pneumatophores per tree N_{pneu}. Pneumatophores are the vertical aerial breathing roots that the trees use to adapt to tidal flooding and the low oxygen supply in sediments. They can grow to a few tens of centimetres high [6] and thus increase the overall flow resistance. A single *Avicennia marina* tree can have more than 10000 of these vertical roots [7]. We developed a sigmoid function which relates N_{pneu} and the stem diameter such that the number of pneumatophores is 10000 for a tree with a stem diameter equal to $D_{\max}$:

$$N_{pneu} = 10025\left(\frac{1}{1+\exp\left[f\left(D_{0.5}-D\right)\right]}\right), \quad (2.3)$$

where f and $D_{0.5}$ are set to 0.3 and 20, respectively. The diameter and height of the pneumatophores are set to 1.0 and 15 cm, respectively.

For the computation of A and V_M in equation (2.1), we simplified the shape of the trunk and the pneumatophores to cylinders and did not consider the crown of the tree, assuming that the crown of the tree is limited in size or above high tide. This assumption follows *Küchler* [6] who described the crown of *Avicennia marina* as well above high tide when the growth of the tree is not limited by low temperatures. Furthermore, we here used the maximum water depth throughout the tidal cycle for the computation of V. To give an example, equation (2.2) implies that for a mangrove density of 125 individuals per ha and trees with a stem diameter of 40 cm, the drag coefficient C_D, also accounting for the pneumatophores, amounts to 1.91, 4.76, and 6.35 for water depths of respectively 0.5, 0.2, and 0.1 m. These C_D-values are within the range of measurements found by Mazda et al. [4,5]. The effect of mangroves on drag can be incorporated in the numerical model as a bottom friction [8,9]. The drag coefficient values computed following equation (2.2) were thus used as input for the bottom friction formulation within the hydrodynamic model and, as such, the mangroves directly affected hydrodynamic conditions.

Sediment transport rates are calculated in the model with the *Engelund and Hansen* [10] formula which is traditionally written as:

$$S_{flow} = \frac{0.05U^5}{\sqrt{g}C^3\Delta^2 D_{50}}, \quad (2.4)$$

where S_{flow} is the sediment transport flux, U is the magnitude of flow velocity, g is the gravitational acceleration, C is the Chézy coefficient (set to 65 $m^{0.5}/s$), Δ is the relative density $(\rho_s-\rho)/\rho$, ρ_s is the sediment density, ρ is the water density, and D_{50} is the median grain size. Equation (2.4) is developed with the assumption that the critical Shields (mobility) parameter θ_{cr} is 0.06. Mangroves increase the resistance of sediments to erosion because the roots of the mangroves play a role in stabilizing the sediments [11]. If we relax the assumption of a constant θ_{cr}, the sediment transport model can be written as:

$$S_{flow} = \frac{0.125CUD_{50}}{\sqrt{g}}\left(0.06 + 0.4\theta^2 - \theta_{cr}\right) \quad \text{for} \quad 0.06 + 0.4\theta^2 > \theta_{cr} \quad \text{and} \qquad (2.5)$$

$$S_{flow} = 0 \quad \text{for} \quad 0.06 + 0.4\theta^2 \leq \theta_{cr}\,, \qquad (2.6)$$

where θ is the Shields parameter which is given by:

$$\theta = \frac{\rho U^2}{C^2(\rho_s - \rho)D_{50}}. \qquad (2.7)$$

Equations (2.5) and (2.6) allow for an erosion threshold which is dependent on the below-ground biomass. No extensive data sets are available to parameterize the effects of *Avicennia marina* on sediment erodibility. We therefore adopted and modified a formulation based on the work by *Mariotti and Fagherazzi* [12], who linearly correlated the increase in erosion threshold with biomass in their model of salt marsh dynamics:

$$\theta_{cr} = \theta_{cr,no}\left(1 + K_{cr}\frac{B_b}{B_{b,mature}}\right), \qquad (2.8)$$

where $\theta_{cr,no}$ is the critical Shields parameter when no mangroves are present and is set to 0.06 such that the sediment transport model reduces to equation (2.4) when mangroves are not present. B_b is the below-ground biomass and $B_{b,mature}$ is the below-ground biomass of 125 mature (D=$D_{\max}$) mangroves. K_{cr} is a constant and we use a value of 0.1 so that θ_{cr} is 10% larger in a mature mangrove forest. Following equations (2.5), (2.6), and (2.7), a 10%-increase (θ_{cr}=0.066) implies that no sediment transport occurs for a flow velocity below 0.31 m/s. Because of the self-thinning process in the mangrove forest, it is possible that during the development of the forest, although the below-ground tree weight per tree is lower, the large number of individuals causes B_b to be higher than $B_{b,mature}$. However, in the model we restrict the ratio between B_b and $B_{b,mature}$ in equation (2.8) so that it cannot exceed 1.

The roots of mangrove trees do not only increase the sediment's resistance to erosion by the tidal flow, they also decrease the magnitude of slope-driven sediment transport fluxes and allow steeper slopes to develop in the morphology. Modelling efforts, which have previously

explored the effects of a reduction in gravitationally driven sediment transport by vegetation [13,14]), applied a reduction by approximately two orders of magnitude in the case of fully developed vegetation. Similar to *Kirwan and Murray* [13], the slope-driven sediment transport S_{slope} is proportional to the bed slope:

$$S_{slope} = \left(\alpha - \beta \frac{B_b}{B_{b,mature}} \right) b, \quad (2.9)$$

where b is the slope towards the neighbouring grid cell. Slope-driven sediment transport only occurs when b exceeds 0.01. This threshold is used so that channel initiation is not hindered and to avoid indefinite channel widening. The ratio between B_b and $B_{b,mature}$ is again not allowed to exceed 1. We here took a conservative approach and applied a maximum reduction of S_{slope} by one order of magnitude and the values of the dimensional constants α (1.1574×10^{-5} m^2/s) and β (1.0417×10^{-5} m^2/s) were chosen accordingly. If mangroves are not present, equation (2.9) reduces to the transport formulation used in van Maanen et al. [15]. Parameterizing the slope-driven sediment transport term is not straightforward and although a similar type of formulation was previously used for salt marsh channels [13], the approach was initially developed for fluvial channels [14]. Further research is clearly needed to test and develop improved representations of gravitationally driven sediment fluxes.

The production of organic material by the mangroves raises the soil surface by a few millimetres per year [16]. Numerical models to study the dynamics of salt marsh systems usually apply a linear relationship between the production of organic matter and biomass [12,17-19]. The main component of organic deposits in mangrove forests is refractory roots with leaf litter playing a secondary role [20]. We therefore related the elevation change due to organic production ΔZ_{org} to the below-ground biomass:

$$\Delta Z_{org} = K_{org} \frac{B_b}{B_{b,mature}}, \quad (2.10)$$

where K_{org} is a characteristic accumulation rate. Little information exists on root volumetric input and detailed measurements of accumulation rates for *Avicennia marina* are unfortunately not available. Field measurements collected for other species suggest that a

wide range of accumulation rates is possible [16]. We decided to set K_{org} to a conservative 1 mm/year.

3. Use of aerial photographs to analyse the effects of mangroves

Although a different type of system from the one simulated in the present study, aerial photos from the Firth of Thames estuary (North Island, New Zealand) show how the spreading of mangroves influenced channel geometry. The Firth of Thames is unusual in that mangrove habitat has rapidly expanded over the last 50 years as a result of catchment deforestation and increased sediment delivery to the estuary [21]. Although it remains difficult to elucidate vegetation effects from aerial photos (and that is why controlled numerical modelling experiment are valuable), figure S2 shows that the geometry of one of the rivers that flows into the estuary has undergone several changes while mangrove growth expanded, including narrowing (and presumably deepening) of the channel and steepening of the channel banks.

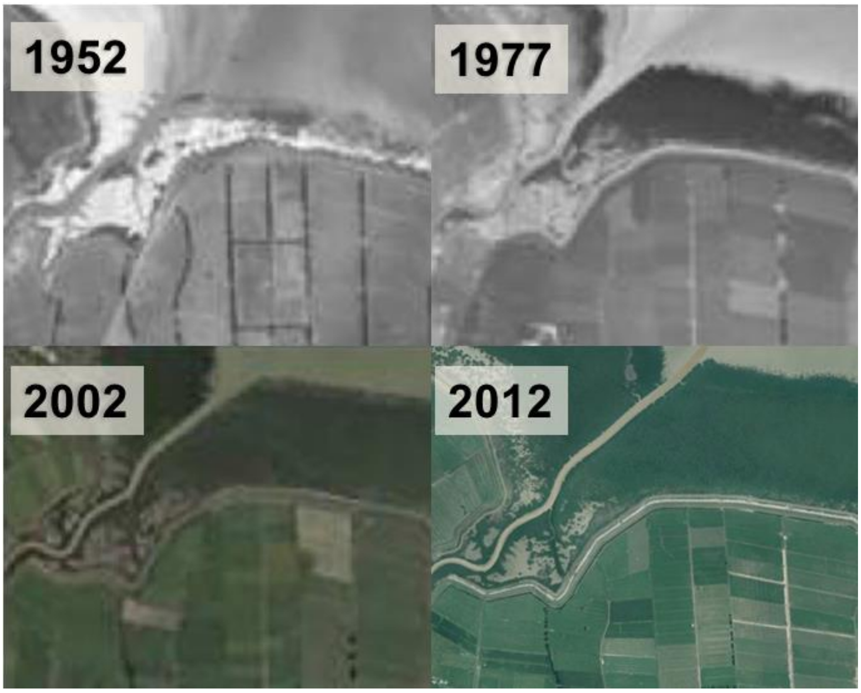

Figure S2. Mangrove expansion and related changes in channel geometry of one of the rivers flowing into the Firth of Thames estuary, North Island, New Zealand.

References

1. Comley BWT, McGuinness KA. 2005 Above- and below-ground biomass, and allometry, of four common northern Australian mangroves. *Aust. J. Bot.* **53**, 431-436. (doi:10.1071/BT04162)
2. Berger U, Hildenbrandt H. 2000 A new approach to spatially explicit modelling of forest dynamics: spacing, ageing and neighbourhood competition of mangrove trees. *Ecological Modelling* **132**, 287-302.
3. Nepf HM. 1999 Drag, turbulence, and diffusion in flow through emergent vegetation. *Water Resour. Res.* **35**, 479-489.
4. Mazda Y, Wolanski E, King B, Sase A, Ohtsuka D, Magi M. 1997 Drag force due to vegetation in mangrove swamps. *Mangroves and Salt Marshes* **1**, 193-199.
5. Mazda Y, Kobashi D, Okada S. 2005 Tidal-scale hydrodynamics within mangrove swamps. *Wetlands Ecol. Managem.* **13**, 647-655. (doi:10.1007/s11273-005-0613-4)
6. Küchler AW. 1972 The mangrove in New Zealand. *New Zealand Geographer* **28**, 113-129.
7. Hogarth PJ. 2007 *The biology of mangroves and seagrasses*, Oxford University Press, Oxford, 273 pp.
8. Mazda Y, Magi M, Kogo M, Hong PN. 1997 Mangroves as a coastal protection from waves in the Tong King delta, Vietnam. *Mangroves and Salt Marshes* **1**, 127-135.
9. Quartel S, Kroon A, Augustinus PGEF, van Santen P, Tri NH. 2007 Wave attenuation in coastal mangroves in the Red River Delta, Vietnam. *J. Asian Earth Sci.* **29**, 576-584. (doi:10.1016/j.jseaes.2006.05.008)
10. Engelund F, Hansen E. 1967 *A Monograph on Sediment Transport in Alluvial Streams*. Teknisk Forlag, Copenhagen, 62 pp.
11. Spenceley AP. 1977 The role of pneumatophores in sedimentary processes. *Mar. Geol.* **24**, M31-M37.
12. Mariotti G, Fagherazzi S. 2010 A numerical model for the coupled long-term evolution of salt marshes and tidal flats. *J. Geophys. Res.* **115**, F01004. (doi:10.1029/2009JF001326)
13. Kirwan ML, Murray AB. 2007 A coupled geomorphic and ecological model of tidal marsh evolution. *Proc. Natl. Acad. Sci. USA* **104**, 6118-6122. (doi:10.1073/pnas.0700958104)

14. Murray AB, Paola C. 2003 Modelling the effect of vegetation on channel pattern in bedload rivers. *Earth Surf. Process. Landforms* **28**, 131-143. (doi:10.1002/esp.428)
15. Van Maanen B, Coco G, Bryan KR. 2013 Modelling the effects of tidal range and initial bathymetry on the morphological evolution of tidal embayments. *Geomorphology* **191**, 23-34. (doi: 10.1016/j.geomorph.2013.02.023)
16. McKee, KL. 2011 Biophysical controls on accretion and elevation change in Caribbean mangrove ecosystems. *Estuarine Coastal Shelf Sci.* **91**, 475-483. (doi:10.1016/j.ecss.2010.05.001)
17. D'Alpaos A, Lanzoni S, Marani M, Rinaldo A. 2007 Landscape evolution in tidal embayments: Modeling the interplay of erosion, sedimentation, and vegetation dynamics. *J. Geophys. Res.* **112**, F01008. (doi:10.1029/2006JF000537)
18. Mudd SM, Fagherazzi S, Morris JT, Furbish DJ. 2004 Flow, sedimentation, and biomass production on a vegetated salt marsh in South Carolina: Toward a predictive model of marsh morphologic and ecologic evolution. In *The Ecogeomorphology of Tidal Marshes, Coastal Estuarine Stud.* (eds Fagherazzi S, Marani M, Blum LK), vol. 59, pp. 165-188, AGU, Washington, D.C.
19. D'Alpaos A, Lanzoni S, Mudd SM, Fagherazzi S. 2006 Modeling the influence of hydroperiod and vegetation on the cross-sectional formation of tidal channels. *Estuarine Coastal Shelf Sci.* **69**, 311-324. (doi:10.1016/j.ecss.2006.05.002)
20. Middleton BA, McKee KL. 2001 Degradation of mangrove tissues and implications for peat formation in Belizean island forests. *J. Ecol.* **89**, 818-828.
21. Lovelock CE, Sorrell BK, Hancock N, Hua Q, Swales A. 2010 Mangrove forest and soil development on a rapidly accreting shore in New Zealand. *Ecosystems* **13**, 437-451. (doi:10.1007/s10021-010-9329-2)
